# Enhancing predictions of subclinical cardiac dysfunction in SLE patients through integrative machine learning analysis

**DOI:** 10.1136/lupus-2025-001616

**Published:** 2025-09-01

**Authors:** Yuhong Liu, Siwei Xie, Zhiming Lin, Changlin Zhao

**Affiliations:** 1 Department of Rheumatology and Immunology, Third Affiliated Hospital of Sun Yat-Sen University, Guangzhou, China; 2 Johns Hopkins Bloomberg School of Public Health, Baltimore, Maryland, USA; 3 Johns Hopkins University School of Medicine, Baltimore, Maryland, USA; 4 Department of Rheumatology, Third Affiliated Hospital of Sun Yat-Sen University, Guangzhou, China; 5 Department of Cardiovascular, Third Affiliated Hospital of Sun Yat-Sen University, Guangzhou, China

**Keywords:** Systemic Lupus Erythematosus, Autoantibodies, Cardiovascular Diseases

## Abstract

**Objective:**

To investigate the two-dimensional speckle-tracking echocardiography (2D-STE) parameters associated with early impaired left ventricular systolic function in SLE patients and to estimate the potential clinical factors that may trigger and influence left ventricular systolic dysfunction.

**Methods:**

This study collected a total of 36 patients admitted to the rheumatology and immunology department of Sun Yat-sen University between January 2020 and December 2021, who were newly diagnosed with SLE and had a Systemic Lupus Erythematosus Disease Activity Index 2000 Score≥4 points. An equal number of healthy controls matched for gender and age were included. All participants underwent routine echocardiography and two-dimensional speckle-tracking echocardiography (2D-STE) examinations. Various clinical data were also collected. Machine learning and regressions were used to estimate potential risk factors for left ventricular systolic dysfunction in SLE patients.

**Results:**

Significant differences in 2D-STE parameters were found, including global longitudinal peak systolic strain (GLPS) (p-adjust<0.001), GLPS strain obtained from the apical two-chamber view and GLPS strain obtained from the apical four-chamber view (GLPS-A4C) (p-adjust=0.005), and GLPS strain obtained from the apical long-axis view (GLPS-APLAX) (p-adjust=0.003) between SLE patients and controls. Machine learning models, particularly GLPS-APLAX, showed excellent discrimination ability with an AUC of 0.93 (95% CI: 0.89 to 0.96) and an area under the precision-recall curve of 0.96. Multivariate regression further highlighted the inverse relationship between anti-U1 small nuclear ribonucleoprotein (U1RNP) antibodies and four GLPS-related continuous variable measures, with GLPS, GLPS-A4C and GLPS-APLAX measures having statistically significant effects (eg, GLPS coefficient=−3.71, 95% CI: −5.91 to −1.51, p=0.002).

**Conclusions:**

This case-control study revealed that 2D-STE parameters can be used to predict subclinical cardiac dysfunction in SLE patients, and anti-U1RNP antibodies may be an essential predictive clinical factor. Machine learning may further assist in preliminary screening and quantifying left ventricular systolic dysfunction reasons in SLE patients.

**WHAT IS ALREADY KNOWN ON THIS TOPIC**

- SLE patients are at high risk for cardiovascular diseases, two-dimensional speckle-tracking echocardiography (2D-STE) and global longitudinal strain are more sensitive for detecting subclinical heart dysfunction in SLE patients.
- Anti-U1 small nuclear ribonucleoprotein (U1RNP) antibodies are linked to some cardiovascular issues, but their association with left ventricular dysfunction is unclear.

**WHAT THIS STUDY ADDS**

- This study integrates machine learning, specifically eXtreme Gradient Boosting and SHapley Additive exPlanations analysis, to predict heart dysfunction in SLE patients.
- Anti-U1RNP antibodies may serve as significant early predictors of left ventricular dysfunction.

**HOW THIS STUDY MIGHT AFFECT RESEARCH, PRACTICE OR POLICY**

- This study encourages further research combining machine learning with medical imaging in autoimmune diseases.
- Clinically, it suggests routine 2D-STE screening for SLE patients, particularly those with anti-U1RNP antibodies, for early intervention.
- It may lead to updated guidelines recommending 2D-STE as a standard screening tool and protocols for monitoring anti-U1RNP in SLE patients.

## Introduction

 SLE is an autoimmune inflammatory connective tissue disease characterised by the production of autoantibodies and the formation of immune complexes, involving multiple organs and systems throughout the body. The cardiovascular system is one of the target organs commonly affected by SLE. Cardiac involvement in SLE occurs at a relatively high rate and can happen at any stage of the disease. Also, as the disease progresses over time, the incidence of cardiac involvement gradually increases. A previous meta-analysis has shown that compared with the general population, patients with SLE are more prone to developing cardiovascular diseases, and this tendency is widely observed worldwide.^1^ Despite cardiac involvement being one of the leading causes of increased mortality and poor prognosis in SLE patients, in reality, due to the lack of apparent symptoms in the early stages of disease progression, cardiac abnormalities such as early myocardial structural changes and left ventricular systolic dysfunction may not be detected through laboratory tests or routine echocardiography examinations.^2^

Currently, there are several echocardiographic techniques and parameters available in clinical practice for evaluating cardiac function. M-mode echocardiography, as well as two-dimensional or three-dimensional echocardiographic measurements, can be used to assess overall left ventricular function. In current clinical practice, left ventricular ejection fractions (LVEF) is the most commonly used index for evaluating left ventricular systolic function due to its simplicity and accessibility.^3^ However, early manifestations of left ventricular systolic dysfunction in patients with SLE may not be apparent. LVEF may still fall within the normal range and may not change easily with disease progression. When changes occur in LVEF, the decline in left ventricular systolic function caused by the disease has already become uncontrollable. Therefore, there is an urgent need in clinical practice for markers that can more accurately and earlier detect changes in left ventricular systolic function for early diagnosis and intervention. With the continuous advancement of echocardiographic technology, novel ultrasound imaging techniques and related parameters have been introduced into the assessment of cardiac function. Following LVEF, myocardial longitudinal strain (LS) is considered one of the most promising indicators in the clinical evaluation of cardiac function.^4 5^ Strain describes the morphological changes that occur in the myocardium relative to its original shape during cardiac contraction. LS refers to the degree of deformation of the left ventricular myocardium during contraction and relaxation movements along the longitudinal axis.

Two-dimensional speckle tracking echocardiography (2D-STE) is an emerging and advanced echocardiographic technique used for evaluating cardiac function. By using computer algorithms to track speckles or markers in consecutive cardiac images, it provides information on myocardial motion trajectories and deformation. 2D-STE offers more accurate and comprehensive information on myocardial motion compared with traditional two-dimensional echocardiography, making it more sensitive and quantitative. It is commonly used in clinical practice for evaluating overall cardiac function, detecting regional functional abnormalities and diagnosing and monitoring cardiac diseases. In addition to analysing LS in individual segments of the left ventricle, 2D-STE can also derive the average longitudinal peak strain of all myocardial segments, known as the global longitudinal strain (GLS). Several studies have demonstrated the superior capability of GLS over LVEF in assessing subclinical cardiac dysfunction and prognostication across diverse pathological conditions. In fact, GLS has demonstrated the potential to enhance the predictive value of adverse outcomes attributable to cardiac dysfunction, thereby surpassing LVEF to some extent.^6^,^8^

The aim of this study was to investigate the 2D-STE parameters associated with early left ventricular systolic dysfunction in SLE patients, employ machine learning techniques to further analyse the correlation with relevant clinical indicators and predict the risk factors that may provoke and influence the left ventricular systolic dysfunction.

## Methods

### Study population

This study recruited a cohort of 45 patients diagnosed with SLE for the first time, who were hospitalised at the rheumatology and immunology department of the Third Affiliated Hospital of Sun Yat-sen University between January 2020 and December 2021. Disease activity was assessed using the Systemic Lupus Erythematosus Disease Activity Index 2000 (SLEDAI-2K), which was published in 2002. Four levels of disease activity were classified according to the scores: 0–4 points, 5–9 points, 10–14 points and ≥15 points, which corresponded to the condition being essentially inactive, mildly active, moderately active and severely active, respectively. After excluding patients with essentially inactive disease, 36 patients were ultimately enrolled in this study. Concurrently, 36 healthy controls without underlying diseases and who underwent the same echocardiography examination at our physical examination centre were included, matched based on gender and age.

All cases required exclusion of patients with structural heart disease, coronary artery disease, valvular heart disease, pericarditis, arrhythmias, heart failure and patients with LVEF<50%. Inclusion criteria are as follows: (1) patients diagnosed with SLE for the first time must meet the 2019 European League Against Rheumatism/American College of Rheumatology classification criteria for SLE; (2) age range needed to be between 18 and 65 years old; (3) laboratory examinations for heart failure indicators such as brain natriuretic peptide, myocardial biomarkers such as troponin, creatine kinase (CK) and CK isoenzymes need to remain within normal ranges; (4) no significant abnormalities in ECG and X-ray of the chest. Exclusion criteria include: (1) pregnancy; (2) known presence of severe hepatic or renal insufficiency; (3) presence of other severe systemic diseases such as malignancy, other autoimmune diseases, etc; (4) History of cardiac surgery or other surgical interventions interfering with the assessment of cardiac function.

### Left ventricular segmentation

**Figure 1:**
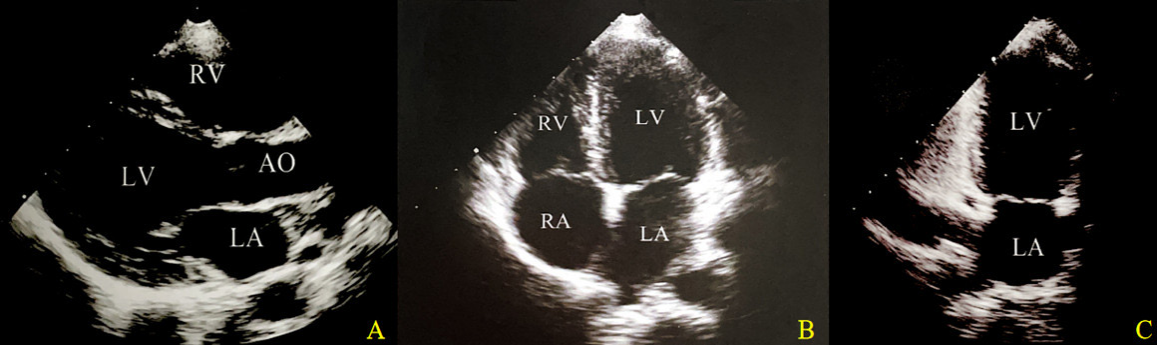
Schematic division of the left ventricular section. (A) Apical parasternal long-axis view. (B) Apical four-chamber view. (C) Apical two-chamber view. AO, aorta; LA, left atrium; LV, left ventricle; RA, right atrium; RV, right ventricle.

The segmentation of the left ventricular with reference to standardised ventricular segmentation, nomenclature and coronary region allocation methods developed by the American Heart Association and the European Society of Cardiology.^9^ In echocardiography, the left ventricle is divided into three planes: short axis, vertical long axis and horizontal long axis. These correspond to the apical parasternal long-axis (APLAX), apical four-chamber (A4C) and apical two-chamber (A2C) views, respectively (figure 1). The most commonly used method for segmenting the left ventricular myocardium is based on these 3 views, further subdivided into 17 segments according to the 3 myocardial rings of the left ventricle.

Assessment of left ventricular segmental motion is performed by measuring the overall LS of each segment corresponding to APLAX, A4C and A2C views. Following 2D-STE measurements, three prevalent strain parameters used for assessing left ventricular systolic function are acquired: global longitudinal peak systolic strain obtained from the apical long-axis view (GLPS-APLAX), global longitudinal peak systolic strain obtained from the apical four-chamber view (GLPS-A4C), and global longitudinal peak systolic strain obtained from the apical two-chamber view (GLPS-A2C). The global longitudinal peak systolic strain (GLPS or GLS) of the left ventricle is defined as the peak strain value of the averaged strain curves from the three apical views. In addition to measuring GLPS, GLPS-APLAX, GLPS-A4C and GLPS-A2C, peak systolic strain is also measured for each of the 17 segments corresponding to the systolic peak strain of each segment. Reference ranges for all echocardiographic parameters are based on guidelines from the American Society of Echocardiography (ASE) for transthoracic echocardiography and 2D-STE.

### Definition of GLS reference values

Given the lack of large-sample, multicentre clinical research data in China, there is currently no unified standard for normal values and reference ranges of GLS in the Chinese population. The widely used normal reference value threshold remains GLS≤ −20% as recommended by the 2015 ASE guidelines.^10^ A multicentre clinical study conducted worldwide by the World Alliance Societies of Echocardiography (WASE) in 2019 provided normal ranges for left ventricular global longitudinal strain (LVGLS) across different genders, ethnicities and countries. It illustrated that GLS is correlated with gender and age, with slightly higher absolute values in females and decreasing values with increasing age. The study results from WASE suggested that in the Chinese population, LVGLS< −17% in males and LVGLS< −18% in females indicate impaired left ventricular systolic function. However, due to significant differences in GLS measurements obtained from instruments from different manufacturers, and the inherent measurement errors and selection bias in the strain data analysed, which are provided by the vendors, it is difficult to avoid biases.^11^ Therefore, our study still adopts −20% as the standard reference threshold for assessing whether left ventricular systolic function is impaired, and simultaneously uses it as the standard for assessing the normal ranges of GLPS-APLAX, GLPS-A4C and GLPS-A2C.

### Data collection

All study participants underwent routine echocardiography and 2D-STE examinations performed by an experienced cardiac sonographer. Routine left ventricular systolic function parameters were obtained, including left ventricular end-diastolic diameter, end-diastolic volume, stroke volume, cardiac output (CO), ejection fraction (EF), fractional shortening and aortic valve closure time, as well as left ventricular segmental motion-related strain parameters, respectively. In addition, baseline clinical data of SLE patients were collected, including gender, age, SLEDAI Score, systemic involvement and laboratory test results such as complete blood count, biochemistry blood test, lipid profile, complement test, renal function and autoantibody indices.

### Machine learning prediction modelling

In this case–control study, we designed an explainable machine learning model focusing on echocardiographic outcomes pertinent to SLE. To facilitate machine learning analyses, the continuous variables GLPS, GLPS-A2C, GLPS-A4C and GLPS-APLAX were binarised, streamlining the classification process. A broad array of predictors encompassing demographic and clinical baseline data was incorporated into the machine learning models (online supplemental Table S1). We trained the models using 70% of the dataset, with a 30% holdout dataset reserved for both testing and evaluation. The holdout dataset did not include in any training part, which made sure that performance metrics would derive exclusively from a truly independent test set, mitigating the risk of overly optimistic estimates. We also acknowledge, however, that due to resource constraints and study design limitations, external validation was not conducted in this study, which may limit the generalisability of the proposed model to some extent.

As the size of our dataset was relatively small, we also adopted resampling techniques as a method to further balance the dataset. We employed the robust eXtreme Gradient Boosting (XGBoost) algorithm,^12^ depending on its great ability in handling sparse and complex data structures inherent to multidimensional datasets. We normalised continuous variables (which were in different ranges) to the same range before training the model. Categorical variables were encoded using one-hot encoding prior to model training. For the model hyperparameters, we rigorously tuned tree depth (optimal value=3), minimal node size (optimal value=5) and mtry (optimal proportion=0.8) to optimise predictive performance. Other hyperparameters were maintained at their default settings. We optimised through a 10-fold cross-validation combined with 15-fold grid search. Model performance was tested using area under the receiver operating characteristic curve (AUC), the area under the precision-recall curve (AUPRC) and the Hosmer-Lemeshow Test (for calibration) with calibration curve.^13 14^ We also calculated the 95% CI to show the uncertainty for AUC values. Calibration analysis was conducted to test the agreement between predicted probabilities and actual event rates.

To further explain our model, we also applied the SHapley Additive exPlanations (SHAP) analysis.^15^ The SHAP importance value was instrumental in interpreting the model, quantifying the influence of each predictor variable and providing a granular understanding of their impact on the model’s output. We added the model performance metrics only for the top 10 variables from SHAP analysis. This feature selection helps mitigate overfitting, reduces model complexity and allows us to evaluate the potential for model simplification while retaining predictive value.

### Statistical analysis

#### Sample size

Due to the exploratory nature of this pilot study and the absence of prior similar research, a formal power calculation was not feasible. Instead, we retrospectively assessed our sample size using the events per variable (EPV) guideline widely recommended for multivariable predictive modelling. The commonly accepted minimum standard is 10 EPV to reliably avoid overfitting. Initially, our exploratory model included many candidate variables, resulting in an EPV far below the recommended threshold. Recognising this limitation, we explicitly employed SHAP analysis-based feature selection, identifying the top 10 predictors, and subsequently developed a simplified predictive model based solely on these 10 variables, increasing the EPV ratios. But our cohort still did not satisfy short of this ideal threshold. To mitigate associated overfitting concerns and potential instability, we adopted various modelling strategies including a rigorous cross-validation scheme with repeated resampling, and SHAP analysis-based feature selection, as we mentioned above. We clearly acknowledge that despite these efforts, our results remain preliminary and future validation studies using larger cohorts with adequate EPV ratios are required to confirm our findings.

Considering the data missingness, missing data were handled by Random Forest-based imputation (missForest). After the machine learning part, we then used multivariate regression analyses to substantiate the findings from the machine learning model and to elucidate the relationships within the data, with the application of the Benjamini-Hochberg correction on p values derived from the regression to account for multiple testing and control the false discovery rate.^16^ We also again calculated the 95% CI to show the coefficient uncertainty. XGBoost modelling and SHAP analysis used R packages xgboost and shapviz, respectively. All analytical procedures were executed using R software, V.4.4.2 (R Project for Statistical Computing).

## Results

We provided a comprehensive comparison of demographic data, general left ventricular function parameters and 2D-STE-related left ventricular strain parameters between the SLE group and the control group (table 1). In the demographics section, age and gender distribution showed no significant difference between the groups. We observed that under the premise of both groups having LVEF greater than 50%, there were no significant differences between the general left ventricular function parameters of the two groups. From the 2D-STE parameters, we found statistically significant differences in GLPS (p-adjust<0.001), GLPS-A2C (p-adjust=0.005), GLPS-A4C (p-adjust=0.005) and GLPS-APLAX (p-adjust=0.003) between the SLE and control groups, with the SLE group exhibiting reduced strain values.

**Table 1:** Variables comparison between SLE and control group (mean±SD)

Variable	SLE (n=36)	Control (n=36)	P value	P-adjust
**Demographics**				
Age	34.22 (±13.75)	35.11 (±14.05)	0.795	0.986
Gender Female (n%)	34 (94.44%)	34 (94.44%)	1.000	1.000
**General left ventricular function parameters**				
LVIDd (mm)	43.87 (±4.79)	44.00 (±2.96)	0.453	0.702
HR (bpm)	82.44 (±9.82)	81.42 (±14.44)	0.347	0.566
EDV (ml)	88.67 (±24.03)	88.64 (±13.08)	0.324	0.558
SV (ml)	59.52 (±12.72)	60.56 (±10.00)	0.701	0.905
CO (L/min)	4.93 (±1.31)	4.91 (±1.11)	0.996	1.000
EF (%)	68.48 (±7.01)	68.81 (±4.93)	0.819	0.976
FS (%)	38.17 (±6.06)	38.56 (±4.12)	0.892	1.000
AVC (ms)	332.81 (±27.19)	342.94 (±25.11)	0.137	0.250
**2D-STE-related left ventricular strain parameters**				
GLPS-APLAX (%)	−18.80 (±3.34)	−20.99 (±1.61)	0.001	0.003
GLPS-A4C (%)	−18.91 (±3.67)	−21.09 (±1.31)	0.002	0.005
GLPS-A2C (%)	−19.15 (±3.89)	−21.46 (±1.49)	0.002	0.005
GLPS (%)	−19.01 (±3.12)	−21.19 (±1.19)	<0.001	<0.001
BS Peak LS (%)	−14.32 (±5.37)	−20.88 (±1.91)	<0.001	<0.001
BAS Peak LS (%)	−15.04 (±4.81)	−20.36 (±1.70)	<0.001	<0.001
MS Peak LS (%)	−18.98 (±3.31)	−20.73 (±1.65)	0.007	0.016
MAS Peak LS (%)	−18.92 (±3.77)	−20.69 (±2.09)	0.029	0.060
AS Peak LS (%)	−24.08 (±5.51)	−24.14 (±3.50)	0.604	0.851
BA Peak LS (%)	−14.08 (±5.80)	−20.92 (±2.33)	<0.001	<0.001
MA Peak LS (%)	−17.45 (±5.03)	−20.16 (±1.43)	<0.001	<0.001
AA Peak LS (%)	−22.83 (±5.61)	−23.42 (±3.35)	0.960	1.000
BL Peak LS (%)	−14.78 (±4.92)	−20.71 (±1.55)	<0.001	<0.001
ML Peak LS (%)	−17.60 (±3.98)	−20.26 (±1.43)	<0.001	<0.001
AL Peak LS (%)	−23.02 (±5.29)	−22.90 (±2.53)	0.928	1.000
BP Peak LS (%)	−16.12 (±4.88)	−20.98 (±3.11)	<0.001	<0.001
MP Peak LS (%)	−17.44 (±3.86)	−20.27 (±1.59)	<0.001	<0.001
BI Peak LS (%)	−16.89 (±4.35)	−21.93 (±2.98)	<0.001	<0.001
MI Peak LS (%)	−19.25 (±4.99)	−21.60 (±2.63)	0.038	0.074
AI Peak LS (%)	−23.58 (±5.54)	−24.27 (±3.26)	0.524	0.774
AP Peak LS (%)	−23.82 (±5.41)	−24.33 (±2.32)	0.605	0.815

AA, apical anterior segment; AI, apical inferior segment; AL, apical lateral segment; AP, apex segment; AS, apical septal segment; AVC, aortic valve closure; BA, basal anterior segment; BAS, basal anteroseptal segment; BI, basal inferior segment; BL, basal lateral segment; BP, basal inferolateral segment; BS, basal inferoseptal segment; CO, cardiac output; 2D-STE, two-dimensional speckle-tracking echocardiography; EDV, end-diastolic volume; EF, ejection fractions; FS, fractional shortening; GLPS, global longitudinal peak systolic strain; GLPS-A2C, global longitudinal peak systolic strain obtained from the apical two-chamber view; GLPS-A4C, global longitudinal peak systolic strain obtained from the apical four-chamber view; GLPS-APLAX, global longitudinal peak systolic strain obtained from the apical long-axis view; HR, heart rate; LVIDd, left ventricular end-diastolic internal diameter; MA, mid anterior segment; MAS, mid anteroseptal segment; MI, mid inferior segment; ML, mid lateral segment; MP, mid inferolateral segment; MS, mid inferoseptal segment; Peak LS, peak systolic longitudinal strain; SV, stroke volume.

### Stratified differences

**Figure 2:**
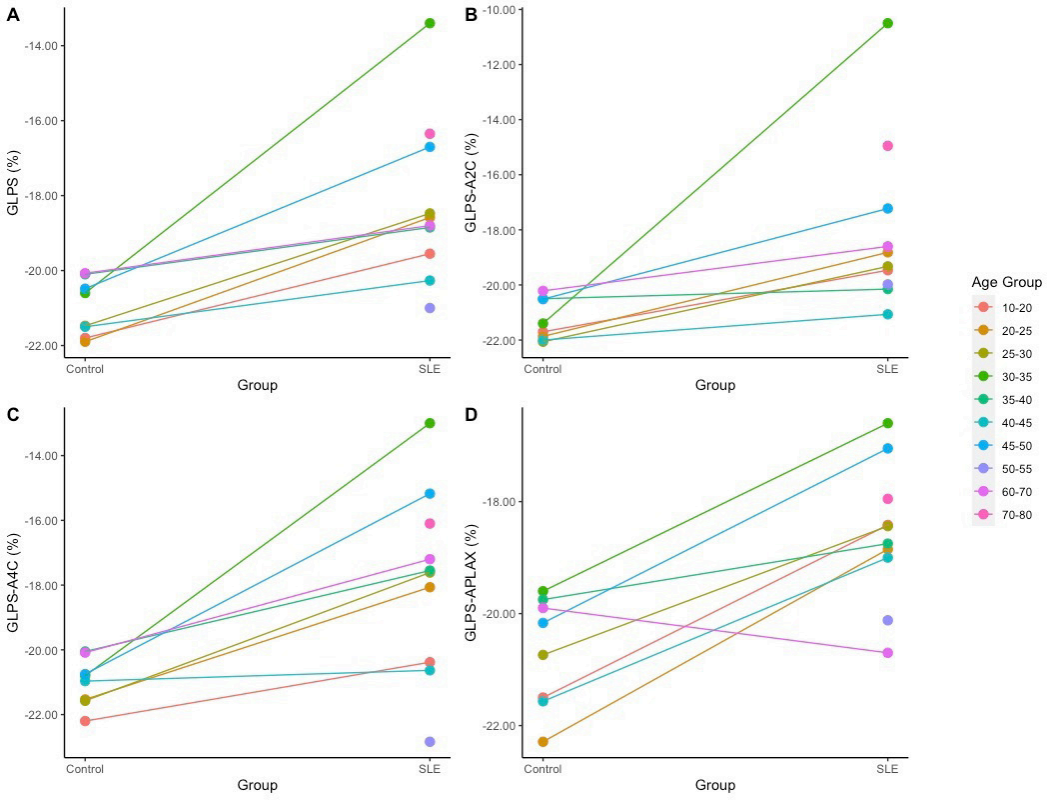
Age-stratified differences between SLE and control group. Each line represents an age group. The x-axis segregates the control and SLE groups. The y-axis quantifies the indicators percentage. GLPS, global longitudinal peak systolic strain; GLPS-A2C, GLPS obtained from the apical two-chamber view; GLPS-A4C, GLPS obtained from the apical four-chamber view; GLPS-APLAX, GLPS obtained from the apical long-axis view.

We displayed the distribution of percentage differences (%) for the four primary metrics between the SLE patients and the control group (online supplemental Figure S1). Values within the healthy control group predominantly concentrate within the normal range, exhibiting a symmetrical distribution with −20% as a demarcation line, suggesting relatively normal left ventricular systolic function. In contrast, values within the SLE group exhibit higher density to the right of the −20% demarcation line, with a notably broader overall range, suggesting a relatively greater prevalence of impaired left ventricular systolic function among SLE patients, accompanied by considerable inter-individual variability. We also concluded the trajectories that illustrated the mean differences between the SLE patients and control subjects across different age groups (figure 2). The lines mapped the progression of mean indicators differences, and we used various colours to represent various age brackets, ranging from 10–20 to 70–80 years.

Due to the influence of age variation on GLS, the variance in observation percentages was not uniform across the age strata. From an overall trend perspective, in most age categories, the results of GLPS, GLPS-A2C, GLPS-A4C and GLPS-APLAX in the SLE group are notably different from those in the healthy control group, with corresponding values significantly greater than −20%. We observed considerable deviations in the age groups of 30–35 years and 45–50 years within the SLE group.

### Feature importance

**Figure 3:**
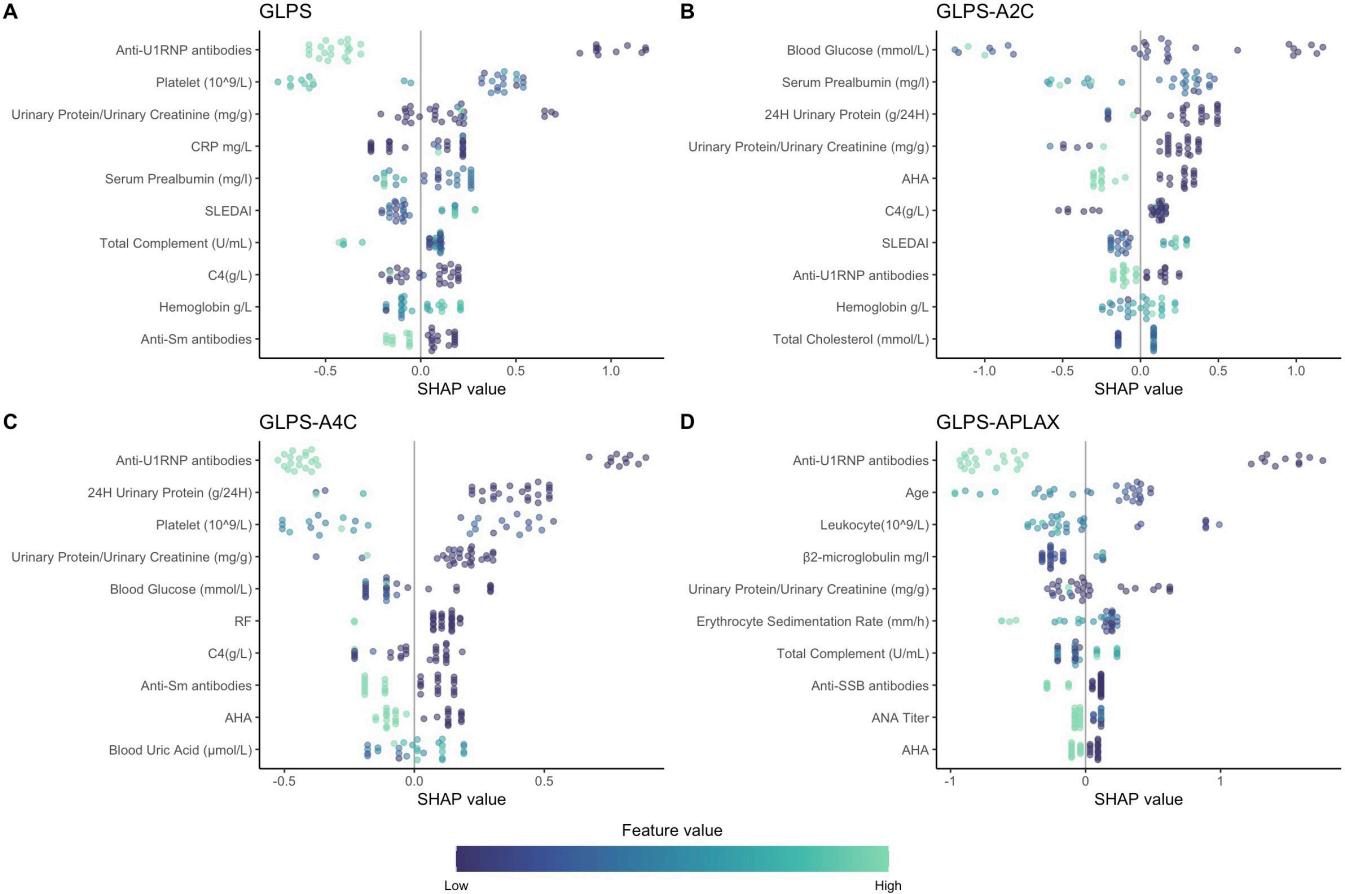
SHAP importance value for XGBoost model predictors. The position on the x-axis reflects the impact of that predictor on the model’s performance: >0 means positive influence and <0 means negative influence. The colour intensity represents the magnitude of value, ranging from low (green) to high (purple). AHA, anti-histone antibodies; ANA, antinuclear antibodies; Anti-Sm, anti-Smith antibodies; Anti-SSB, anti-Sjögren syndrome antigen B; Anti-U1RNP, anti-U1 small nuclear ribonucleoprotein antibodies; CRP, C-reactive protein; GLPS, global longitudinal peak systolic strain; GLPS-A2C, GLPS obtained from the apical two-chamber view; GLPS-A4C, GLPS obtained from the apical four-chamber view; GLPS-APLAX, GLPS obtained from the apical long-axis view; RF, rheumatoid factor; SHAP, SHapley Additive exPlanations; SLEDAI, Systemic Lupus Erythematosus Disease Activity Index; XGBoost, eXtreme Gradient Boosting.

We elucidated the influence of various clinical predictors on the XGBoost machine learning models’ prediction performance by SHAP beeswarm plots (figure 3). SHAP values offered an insightful look into the contribution of each predictor variable. Across all panels (figure 3A–D), the distribution of SHAP values suggests a non-linear and complex relationship between feature values and the impact on the model’s output. In GLPS, the distribution of SHAP values highlighted the substantial influence of anti-U1 small nuclear ribonucleoprotein (U1RNP) antibodies, platelet count, urine protein/creatinine ratio, SLEDAI and C-reactive protein (CRP). Similarly, GLPS-A2C revealed potential influencing factors such as blood glucose, serum prealbumin, anti-histone antibodies, SLEDAI and anti-U1RNP antibodies. The GLPS-A4C was similar to GLPS. Furthermore, in GLPS-APLAX, besides anti-U1RNP antibodies, other top-ranking predictive factors include age, white blood cell count, erythrocyte sedimentation rate (ESR) and anti-Sjögren syndrome antigen B (SSB) antibodies, which are less observed in other SHAP plots. Additionally, we also calculated the average absolute SHAP values for various features (online supplemental Figure S2).

### Multivariate regression

Based on the obvious features in SHAP analysis, we also did multivariate linear regression for four outcomes’ continuous values (table 2). We found a statistically significant inverse association between anti-U1RNP antibodies and many left ventricular function measures: GLPS (coefficient= −3.71 (−5.91 to −1.51), p=0.002), GLPS-A4C (coefficient= −4.22 (−6.83 to −1.61), p=0.003) and GLPS-APLAX (coefficient= −4.53 (−6.61 to −2.45), p<0.001). Platelet count showed a negative but non-significant trend with GLPS-A4C (coefficient= −0.02 (−0.04 to 0.01), p=0.053). SLEDAI was positively correlated with GLPS (coefficient=0.19 (0.03 to 0.36), p=0.030) and GLPS-A4C (coefficient=0.30 (0.12 to 0.49), p=0.003). No significant associations were observed for C4 levels.

**Table 2:** Regression results based SHAP analysis

Variables	Coefficient	SE	95% CI	P value	P-adjust (within each outcome)	P-adjust (among all outcomes)
**GLPS**						
Anti-U1RNP antibodies	−3.71	1.12	−5.91 to −1.51	0.002	0.010	0.020
Platelet	−0.01	0.01	−0.03 to 0.01	0.119	0.198	0.264
Serum prealbumin	<0.01	0.01	−0.01 to 0.02	0.579	0.579	0.643
SLEDAI	0.19	0.09	0.03 to 0.36	0.030	0.075	0.120
C4	−1.00	0.93	−2.83 to 0.83	0.291	0.364	0.416
**GLPS-A2C**						
Anti-U1RNP antibodies	−2.51	1.42	−5.28 to 0.27	0.086	0.430	0.246
Platelet	−0.01	0.01	−0.03 to 0.01	0.344	0.430	0.430
Serum prealbumin	-(<0.01)	0.01	−0.02 to 0.01	0.715	0.715	0.753
SLEDAI	0.15	0.10	−0.05 to 0.35	0.160	0.400	0.267
C4	−1.15	1.08	−3.27 to 0.98	0.297	0.495	0.396
**GLPS-A4C**						
Anti-U1RNP antibodies	−4.22	1.33	−6.83 to −1.61	0.003	0.010	0.017
Platelet	−0.02	0.02	−0.04 to 0.01	0.053	0.088	0.177
Serum prealbumin	0.01	0.01	−0.01 to 0.03	0.250	0.250	0.385
SLEDAI	0.30	0.10	0.12 to 0.49	0.003	0.007	0.015
C4	−1.69	1.08	−3.80 to 0.42	0.126	0.158	0.252
**GLPS-APLAX**						
Anti-U1RNP antibodies	−4.53	1.06	−6.61 to −2.45	<0.001	0.001	0.003
Platelet	−0.01	0.01	−0.03 to 0.01	0.139	0.232	0.253
Serum prealbumin	0.01	0.01	−0.01 to 0.02	0.481	0.601	0.566
SLEDAI	0.15	0.09	−0.03 to 0.33	0.103	0.258	0.258
C4	−0.24	0.97	−2.14 to 1.65	0.804	0.804	0.804

All p-adjust values are adjusted using the Benjamini-Hochberg correction method, which controls the false discovery rate during multiple tests.

Anti-U1RNP, anti-U1 small nuclear ribonucleoprotein; GLPS, global longitudinal peak systolic strain; GLPS-A2C, GLPS obtained from the apical two-chamber view; GLPS-A4C, GLPS obtained from the apical four-chamber view; GLPS-APLAX, GLPS obtained from the apical long-axis view; SHAP, SHapley Additive exPlanations; SLEDAI, Systemic Lupus Erythematosus Disease Activity Index.

### Model performance

**Figure 4:**
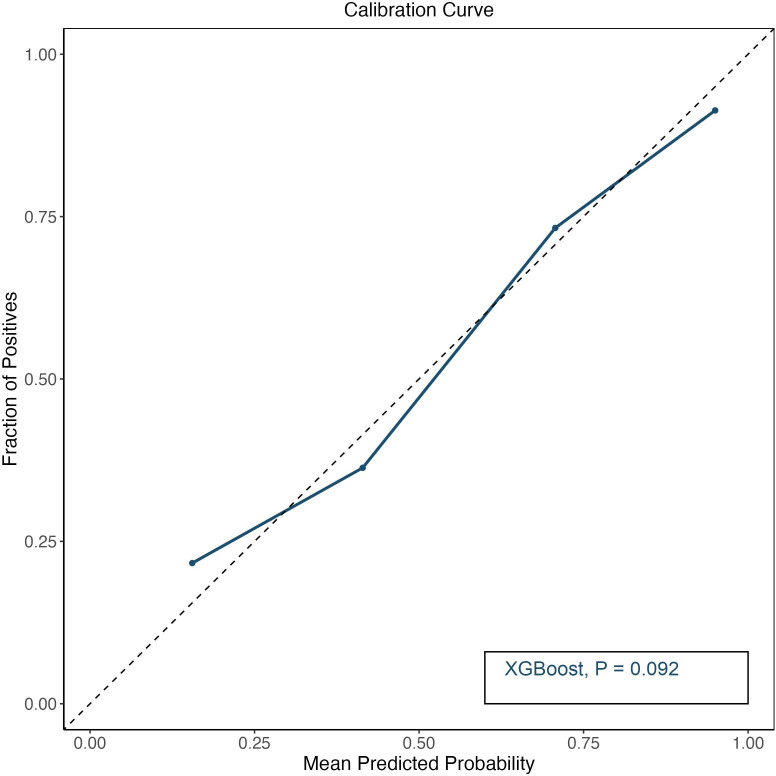
Calibration curve for eXtreme Gradient Boosting (XGBoost) model. P value was obtained from Hosmer-Lemeshow test.

We tested the performance metrics of XGBoost machine learning models developed for the prediction of four cardiac indicators: GLPS, GLPS-A2C, GLPS-A4C and GLPS-APLAX (table 3). The AUC values ranged from 0.70 for GLPS-A4C to 0.93 for GLPS-APLAX. A higher AUC value indicated a better predictive accuracy of correctly discriminating whether SLE patients had cardiac indicator involvement. In contrast, the AUPRC values exhibited more variation, from as low as 0.61 for GLPS-A2C to as high as 0.96 for GLPS-APLAX. The performance of the GLPS-APLAX model was performed great in both AUC (0.93, 95% CI: 0.89 to 0.96) and AUPRC (0.96) scores, indicating a high degree of both discrimination and precision in predicting true positive cases. In addition, except for the GLPS-APLAX model, the performance of the GLPS model also showed high AUC (0.87, 95% CI: 0.82 to 0.92) and AUPRC (0.92). The calibration curve was provided in figure 4, with p value from Hosmer-Lemeshow test>0.05. Model performance metrics only for top 10 variables from SHAP analysis were provided in online supplemental Table S2.

**Table 3:** XGBoost model performance metrics results

Outcome variables	AUC	**AUC** 95% CI	AUPRC
GLPS	0.87	(0.82 to 0.92)	0.92
GLPS-A2C	0.75	(0.70 to 0.80)	0.61
GLPS-A4C	0.70	(0.66 to 0.74)	0.83
GLPS-APLAX	0.93	(0.89 to 0.96)	0.96

Here the GLPS, GLPS-A2C, GLPS-A4C, GLPS-APLAX are all transformed binary variables.

AUC, area under the curve; AUPRC, area under the precision-recall curve; GLPS, global longitudinal peak systolic strain; GLPS-A2C, GLPS obtained from the apical two-chamber view; GLPS-A4C, GLPS obtained from the apical four-chamber view; GLPS-APLAX, GLPS obtained from the apical long-axis view; XGBoost, eXtreme Gradient Boosting.

We also visualised the precision-recall curves for XGBoost models (online supplemental Figure S3). Each panel illustrated a suite of precision-recall curves generated through a resampling methodology (especially, repeated cross-validation) to counteract the limitations imposed by a small dataset, reduced variance and mitigated the risk of overfitting. For the GLPS-A2C indicator and the GLPS-A4C indicator, the models exhibited a broad range of precision values at increasing levels of recall, indicating a larger variability in predictive performance. Conversely, the precision-recall curves for the GLPS-APLAX indicator demonstrated a higher stable level of precision across nearly all recall thresholds.

## Discussion

In this case–control study, the incidence rate of clinical cardiovascular events is higher in patients with SLE compared with the general population. Studies indicate that despite being typically younger, SLE patients have a significantly increased risk of developing cardiovascular disease. Specifically, the incidence rate of cardiovascular events in SLE patients is typically 2–10 times higher than that in the general population.^17^,^19^ The overall incidence rate of cardiovascular events in SLE patients varies depending on the study, population and definitions employed. According to various studies, the incidence rate of cardiovascular events (such as myocardial infarction, stroke, heart failure, etc) in SLE patients can range from 5% to 30%.^20 21^ In recent years, meta-analyses and systematic reviews concerning cardiovascular events in SLE consistently indicate a significantly increased risk of stroke, myocardial infarction and heart failure among SLE patients compared with the general population. This heightened incidence is partly attributable to the direct impact of SLE on the cardiovascular system, as well as the combined effects of common traditional cardiovascular risk factors among patients (such as hypertension, dyslipidaemia, diabetes mellitus, etc) and treatment-related factors.^22 23^ Heart failure refers to the structural and functional changes in the heart caused by various factors, resulting in a decrease in CO and/or filling function, thereby failing to meet the metabolic demands of tissues. This pathological process encompasses the entire spectrum from impaired cardiac pumping function to complete decompensation. Mid-to-late-stage heart failure directly impacts the long-term prognosis of SLE patients. Early diagnosis and intervention for heart failure in SLE patients can significantly improve the quality of life and overall survival of patients.

The most widely used indicator for assessing left ventricular systolic function remains LVEF. However, as previously mentioned, LVEF is less likely to change in the early stages of disease, and a decline in LVEF often indicates disease progression to the mid-to-late stage. Nowadays, there is growing recognition of the limitations of LVEF in evaluating left ventricular systolic function and predicting disease prognosis. In such circumstances, clinical practice more commonly employs 2D-STE to identify abnormal systolic function in patients with normal LVEF. Previous studies have demonstrated the value of 2D-STE in assessing subclinical myocardial dysfunction, hypertrophic cardiomyopathy, ischaemic heart disease, valvular heart disease and in disease diagnosis.^24^,^29^ The strain parameters of 2D-STE reflecting ventricular systolic and diastolic function, such as LVGLS measured in this study, are now considered superior to LVEF in reflecting left ventricular systolic function. GLS, as a measurement and prognostic indicator for heart failure, holds greater value compared with LVEF. Furthermore, GLS can enhance the predictive value of adverse outcomes to some extent beyond LVEF.^30^ Subsequent clinical studies on LVGLS have concluded that LVGLS measurements exhibit stability, good repeatability and high accessibility. They can be used to assess heart failure risk in patients with conditions such as diabetes, hypertension, coronary artery disease and cardiomyopathy. Additionally, LVGLS can stratify heart failure patients with preserved EF, enabling evaluation of their prognosis and treatment efficacy.^31^,^36^

The results of our study indicate that among SLE patients and healthy controls with LVEF values greater than 50%, a higher proportion of SLE patients exhibit abnormalities in GLPS-APLAX, GLPS-A4C, GLPS-A2C and GLPS compared with the healthy control group, with significant statistical differences observed. In patients with SLE, all four parameters mentioned above showed more pronounced decreases and greater deviations from normal thresholds. This further substantiates the presence of varying degrees of left ventricular systolic dysfunction in some SLE patients, even when LVEF is within normal range. These findings are consistent with previous discussions on the ability of 2D-STE to detect subclinical myocardial damage in SLE patients. Furthermore, our study also found a significant decrease in GLS values in the age groups of 30–35 years and 45–50 years compared with other age groups, suggesting that patients in these two age stages exhibit a more pronounced decline in left ventricular systolic function compared with patients in other age stages. This may be related to the increased risk of cardiovascular events associated with ageing, but only these two age groups showed a greater decline in left ventricular systolic function, and the specific mechanisms involved need to be further investigated.

Previous studies have mostly focused on analysing the relationship between SLEDAI and GLS, without analysing potential factors influencing GLPS, GLPS-A2C, GLPS-A4C and GLPS-APLAX. In fact, a year ago we, conducted a preliminary study exploring the application of 2D-STE in assessing early left ventricular systolic dysfunction in patients with SLE. We proposed the role of GLS as an indicator of subclinical myocardial damage in SLE patients and further analysed the relationship between SLEDAI and GLS.^37^ However, a limitation of this study was the inability to correlate with laboratory test results, as not all patients were willing to undergo an additional 2D-STE examination. Therefore, to further investigate potential clinical indicators, we reselected our study subjects, focusing on SLE patients in the active phase of the disease, and employed new analytical methods. Our study further analysed the potential influencing factors affecting left ventricular systolic function through the establishment and prediction based on a machine learning model. The SHAP values output by the XGBoost model demonstrate the ranking of potential predictive factors for different parameters in the models. The online supplemental Figure S2 highlights the absolute advantage of anti-U1RNP antibodies, which exhibited the highest mean absolute SHAP value in the order of importance of the three main parametric prediction models. This underscores the need for further exploration of the role of anti-U1RNP antibodies in cardiac involvement in SLE at a deeper level.

In SLE patients, the positivity rate for anti-U1RNP antibodies is approximately 20%–50%. A multicentre cohort study on connective tissue disease-associated pulmonary arterial hypertension (CTD-APAH) indicated that in China, SLE is the most common underlying disease associated with CTD-APAH.^38^ The research data from the Chinese Lupus Treatment and Research Group indicates that positive anti-U1RNP antibodies are an independent risk factor for the presence of pulmonary arterial hypertension in patients with SLE.^39^ Anti-U1RNP antibodies have been shown to be of significant value in predicting cardiac involvement and pulmonary arterial hypertension in patients with SSc. In our study, anti-U1RNP antibodies emerged as the most prominent predictive factor for GLPS, GLPS-A4C and GLPS-APLAX, highlighting their crucial role in cardiac involvement, particularly left ventricular systolic dysfunction, in SLE patients.

In summary, our study preliminarily integrated echocardiographic and laboratory test indicators using machine learning to identify potential predictive factors for early left ventricular systolic dysfunction in SLE patients. It suggests that anti-U1RNP antibodies emerged as a promising predictive indicator in our limited sample, but these findings should also be interpreted cautiously, given our small cohort and the absence of external validation. The association between anti-U1RNP antibodies and early impaired left ventricular systolic function in patients with SLE may be achieved through immune complex-mediated myocardial injury. Endothelial dysfunction is considered one of the key pathogenic mechanisms underlying cardiovascular events. Multiple studies indicate that patients with SLE possibly already exhibit endothelial cell dysfunction before experiencing cardiovascular events.^40^,^42^ In patients with SLE, elevated levels of serum cell adhesion molecules and other endothelial dysfunction biomarkers, decreased numbers and abnormal function of endothelial progenitor cells, endothelial cell activation mediating the formation of inflammatory mediators, and disrupted endothelial cell damage and repair mechanisms are crucial mechanisms underlying the occurrence of cardiovascular events.^43^,^45^ However, the specific signalling pathways and immune activation pathways involving anti-U1RNP antibodies in myocardial damage still require further research and exploration.

Anti-U1RNP antibodies may serve as a potential predictive factor for early left ventricular systolic dysfunction in patients with SLE. When a high titre of anti-U1RNP antibodies is detected in laboratory tests, it is recommended to perform 2D-STE screening early to assess potential left ventricular dysfunction. Based on the findings of this study, machine learning models have demonstrated significant potential for early screening of cardiac dysfunction. Specifically, clinicians can use the level of anti-U1RNP antibodies as an effective indicator for screening cardiac dysfunction, allowing for early intervention at the onset of the disease. Early identification of patients with left ventricular dysfunction could help reduce the occurrence of cardiovascular events. Furthermore, machine learning prediction models can be integrated with existing clinical diagnostic methods, enhancing decision support for clinicians. For example, based on these models, doctors can tailor treatment plans for patients, such as adjusting immunosuppressive therapy, optimising medication usage or providing personalised lifestyle recommendations, thereby minimising cardiovascular risks and improving overall patient health management.

Long-term use of glucocorticoids is also correlated with an increased risk of cardiovascular disease in SLE. Considering the influence of medication factors, our study selected patients diagnosed with SLE for the first time to analyse. Our study is not the first to combine 2D-STE technology with cardiac involvement in SLE, but we innovatively integrated clinical indicators with echocardiographic parameters, exploring the relationship between autoantibodies and subclinical cardiac dysfunction in SLE patients. Moreover, our findings have been validated across major 2D-STE parameters.

### Limitations

There are several limitations in our study. First, the sample size was relatively small. We acknowledge that the small sample size could impact the generalisability of the model, especially in machine learning, where overfitting is a potential concern. Future studies should validate our findings in larger, multicentre datasets to further improve the model’s stability. Second, performance metrics should be interpreted with caution. Although we attempted to reduce this risk through internal validation and rigorous cross-validation methods, the external validation in a larger cohort remains indispensable and will be important to further validate the model’s generalisability. Third, echocardiographic outcomes were assessed by experienced cardiac sonographers who were not blinded to patient diagnosis, which may introduce potential observer bias. Future studies should incorporate blinding of echocardiographic assessment to enhance the objectivity and reproducibility of outcomes. Fourth, our study only measured systolic strain parameters of the left ventricle, with a focus on early left ventricular systolic dysfunction. However, it is currently unclear whether early cardiac involvement in SLE also affects left ventricular diastolic function or right ventricular function at an early stage. Therefore, future research should expand to include other cardiac functional parameters to further assess the extent of cardiac involvement in SLE patients.

## Conclusion

This case–control study found that compared with healthy individuals, patients with SLE are more prone to subclinical myocardial damage, and the degree of decline in left ventricular systolic function is relatively greater. For SLE patients, we recommend early screening with 2D-STE to assess left ventricular systolic function. Among the strain parameters obtained from 2D-STE, GLPS-APLAX exhibits relatively high reliability in predicting subclinical cardiac dysfunction in SLE patients. Furthermore, machine learning suggests anti-U1RNP antibodies as a potential predictive factor for early left ventricular systolic dysfunction in SLE patients. These initial findings require validation through future studies with larger and more diverse populations to confirm their clinical applicability.

## Supplementary material

10.1136/lupus-2025-001616 online supplemental file 1

## Data Availability

Data are available upon reasonable request.
