# Supplementary material for: Enhancing predictions of subclinical cardiac dysfunction in SLE patients through integrative machine learning analysis
Source: Lupus Sci Med. 2025 Sep 1;12(2):e001616. doi: 10.1136/lupus-2025-001616 (PMC12406935; doi:10.1136/lupus-2025-001616)
Supplement: online supplemental file 1 [file lupus-12-2-s001.docx]

**Supplementary Online Content**

**Enhancing Predictions of Subclinical Cardiac Dysfunction in Systemic Lupus Erythematosus Patients through Integrative Machine Learning Analysis**

**AUTHORS**

Yuhong Liu ^1†^, Siwei Xie ^2†^, Zhiming Lin ^1*^, Changlin Zhao^3*^

^1^Department of Rheumatology, Third Affifiliated Hospital of Sun Yat-Sen University, Guangzhou, China

^2^Johns Hopkins Bloomberg School of Public Health, Baltimore, Maryland, USA

^3^Department of Cardiovascular, Third Affifiliated Hospital of Sun Yat-Sen University, Guangzhou, China

†: Authors contributed equally.

Yuhong Liu and Siwei Xie contributed equally to this work.

**Corresponding author:**

Zhiming Lin, Department of Rheumatology, Third Affifiliated Hospital of Sun Yat-Sen University, Guangzhou, China. Address: 600 Tianhe Rd, Guangzhou, China, 510000. E-mail: [lzm-zj99@163.com](mailto:lzm-zj99@163.com)

Changlin Zhao, Department of Cardiovascular, Third Affifiliated Hospital of Sun Yat-Sen University, Guangzhou, China. Address: 600 Tianhe Rd, Guangzhou, China, 510000. E-mail: 18922102570@163.com

**Supplementary Table S1.**

List of predictor variables used for machine learning models

**Supplementary Table S2.**

Model performance metrics only for top 10 variables from SHAP analysis

**Supplementary Figure S1.**

2D-STE Main Parameters Distributions (%) in SLE and Control Group

**Supplementary Figure S2.**

SHAP Importance (Mean Absolute Score Ranking)

**Supplementary Figure S3.**

Precision-Recall Curves (Resampling) for XGBoost Models

| **Supplementary Table S1. List of predictor variables used for machine learning models** | | | |
| --- | --- | --- | --- |
| **Types** | **Variable** | **Unit(s)/Class** | **Observation** |
| **Demographic characteristics** | Gender | binary | Female: 94.44%  (n = 34/36) |
|  | Age | year | 34.22 (±13.75) |
| **Disease activity status** | SLEDAI | / | 12.75 (±6.54) |
|  | C-reactive Protein | mg/L | 13.47 (±33.39) |
|  | Erythrocyte Sedimentation Rate | mm/h | 40.26 (±29.66) |
| **Complete Blood Cell Count** | Leukocyte | 10^9/L | 5.80 (±2.70) |
|  | Hemoglobin | g/L | 102.04 (±28.62) |
|  | Platelet | 10^9/L | 205.97 (±81.13) |
| **Biochemistry Blood Test** | Albumin | g/L | 34.89 (±6.16) |
|  | Serum Prealbumin | mg/L | 192.00 (±78.15) |
|  | Blood Glucose | mmol/L | 6.17 (±3.03) |
| **Renal Function Test** | Creatinine | μmol/L | 67.49 (±36.96) |
|  | Blood Uric Acid | μmol/L | 360.18 (±96.22) |
|  | Cystatin C | mg/L | 1.26 (±0.58) |
| **Blood Lipid** **Profile** | Total Cholesterol | mmol/L | 4.86 (±1.70) |
|  | Triglycerides | mmol/L | 1.77 (±1.04) |
|  | High-density Lipoprotein Cholesterol | mmol/L | 1.01 (±0.49) |
|  | Low-density Lipoprotein Cholesterol | mmol/L | 2.93 (±1.20) |
| **Complement Test** | Complement 3 | g/L | 0.65 (±0.34) |
|  | Complement 4 | g/L | 0.24 (±0.67) |
|  | Total Complement | U/mL | 27.01 (±21.21) |
| **Proteinuria Test** | 24 Hour Urinary Protein | g/24H | 2.99 (±5.85) |
|  | Urine Protein / Creatinine Ratio | mg/g | 2707.82 (±6110.65) |
| **Autoantibody Test** | Antinuclear Antibodies | binary | 91.67%  (n= 33/36) |
|  | Anti-dsDNA Antibodies | binary | 75.00%  (n= 27/36) |
|  | Anti-C1q Antibodies | binary | 52.78%  (n= 19/36) |
|  | Anti-nucleosome Antibodies | binary | 44.44%  (n= 16/36) |
|  | Anti-histone Antibodies | binary | 47.22%  (n= 17/36) |
|  | Anti-Sm Antibodies | binary | 41.67%  (n= 15/36) |
|  | Anti-U1RNP Antibodies | binary | 58.33%  (n= 21/36) |
|  | Anti-SSA Antibodies | binary | 63.89%  (n= 23/36) |
|  | Anti-SSB Antibodies | binary | 33.33%  (n= 12/36) |
|  | Anti-Scl-70 Antibodies | binary | 16.67%  (n= 6/36) |
|  | Anti-Jo-1 Antibodies | binary | 16.67%  (n= 6/36) |
|  | Anti-ribosomal P Antibodies | binary | 25.00%  (n= 9/36) |
|  | Anti-PCNA Antibodies | binary | 2.78%  (n= 1/36) |
|  | Anticardiolipin | binary | 16.67%  (n= 6/36) |
|  | PT-IgM | binary | 52.78%  (n= 19/36) |
|  | PT-IgG | binary | 38.89%  (n= 14/36) |
|  | Anti-β2GP1 Antibodies | binary | 22.22%  (n= 8/36) |
|  | Coombs Test | binary | 2.78%  (n= 1/36) |
|  | Rheumatoid Factor | binary | 5.56%  (n= 2/36) |
|  | Anti-keratin Antibodies | binary | 2.78%  (n= 1/36) |
|  | Anti-RA33 Antibodies | binary | 11.11%  (n= 4/36) |
|  | Anti-CCP Antibodies | binary | 2.78%  (n= 1/36) |

| **Supplementary Table S2.** Model performance metrics only for top 10 variables from SHAP analysis | | | |
| --- | --- | --- | --- |
| **Outcome Variables** | **AUC** | **AUC 95% CI** | **AUPRC** |
| GLPS | 0.85 | (0.81 to 0.89) | 0.87 |
| GLPS-A2C | 0.76 | (0.69 to 0.83) | 0.65 |
| GLPS-A4C | 0.72 | (0.67 to 0.77) | 0.81 |
| GLPS-APLAX | 0.89 | (0.85 to 0.93) | 0.90 |
| AUC = Area Under the Curve. AUPRC = Area Under the Precision-Recall Curve. Here the GLPS, GLPS-A2C, GLPSA4C, GLPS-APLAX are all transformed binary variables. | | | |


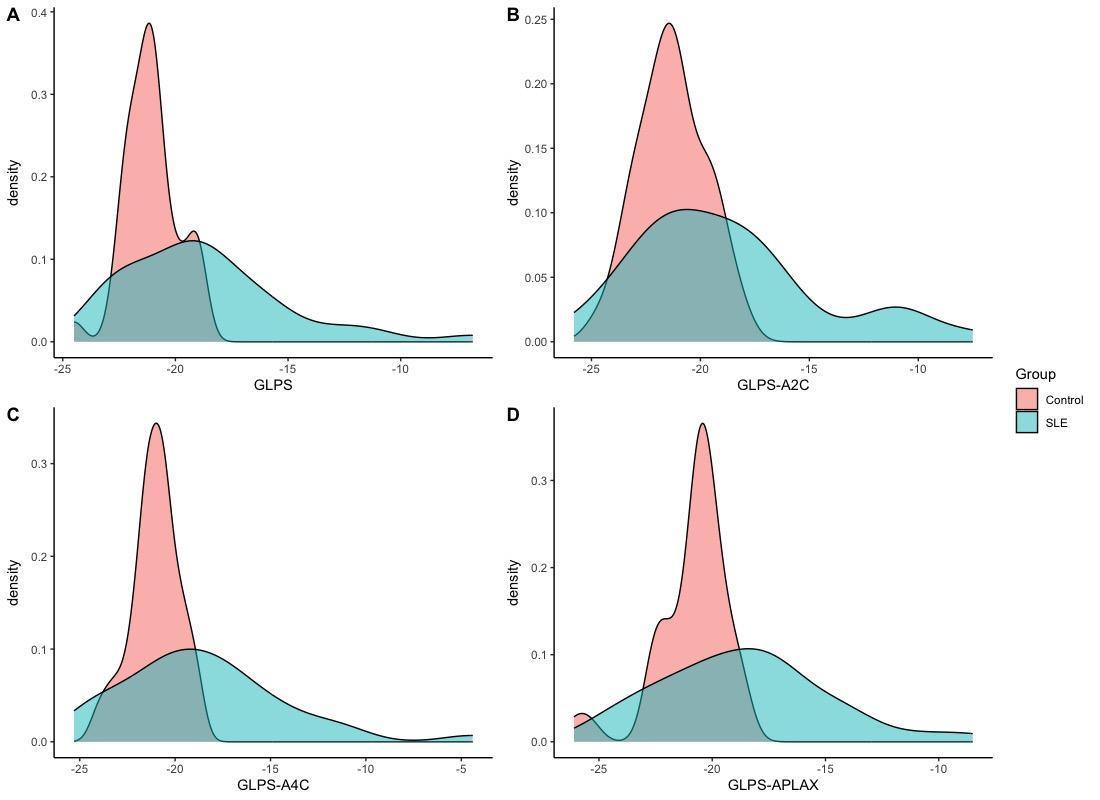


**Supplementary Figure S1.** 2D-STE Main Parameters Distributions (%) in SLE and Control Group. The teal (blue-green) color represents the SLE patient group, while the salmon pink color indicates the control group. The x-axes are labeled GLPS, GLPS-A2C, GLPS-4AC, and GLPS-APLAX for the respective graphs A to D.


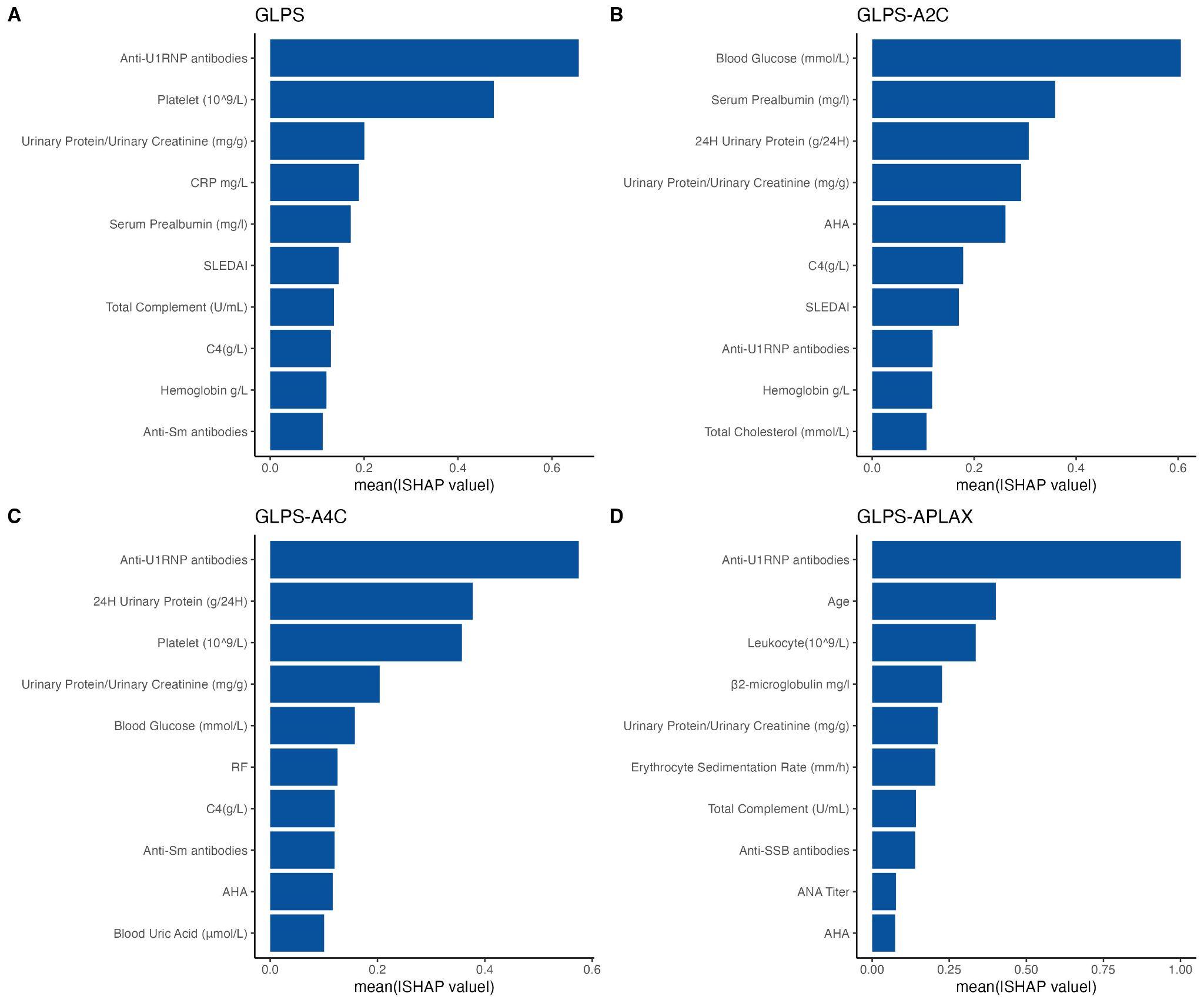


**Supplementary Figure S2.** SHAP Importance (Mean Absolute Score Ranking). Each bar signifies the average absolute impact of a predictor variable on the model's output, with the length representing the magnitude of the mean absolute SHAP value.


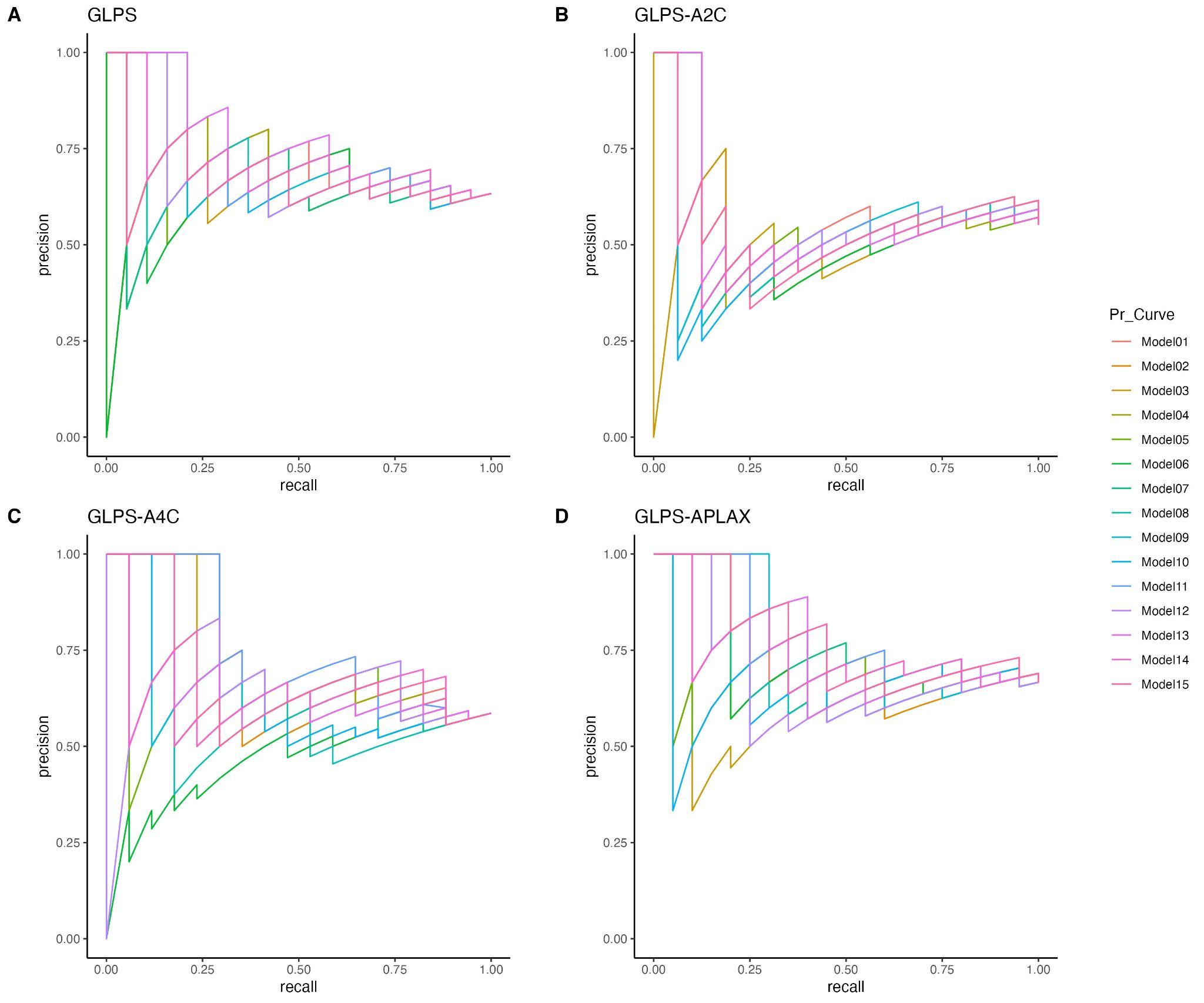


**Supplementary Figure S3.** Precision-Recall Curves (Resampling) for XGBoost Models. Four different cardiac indicators: GLPS (A), GLPS-A2C (B), GLPS-A4C (C), and GLPS-APLAX (D) in SLE patients. Each curve represented a resampled subset of the data, with fifteen iterations per indicator. The x-axis measures recall (true positive rate), and the y-axis measures precision (positive predictive value). The different colors corresponded to individual models built from resampled data sets.
